# Reservoir computing using self-sustained oscillations in a locally connected neural network

**DOI:** 10.1038/s41598-023-42812-9

**Published:** 2023-09-19

**Authors:** Yuji Kawai, Jihoon Park, Minoru Asada

**Affiliations:** 1 Symbiotic Intelligent Systems Research Center, Institute for Open and Transdisciplinary Research Initiatives, Osaka University, Suita, Osaka 565-0871 Japan; 2 Center for Information and Neural Networks, National Institute of Information and Communications Technology, Suita, Osaka 565-0871 Japan; 3 International Professional University of Technology in Osaka, Kita-ku, Osaka 530-0001 Japan; 4 Chubu University Academy of Emerging Sciences, Chubu University, Kasugai, Aichi 487-8501 Japan

**Keywords:** Computational science, Computer science

## Abstract

Understanding how the structural organization of neural networks influences their computational capabilities is of great interest to both machine learning and neuroscience communities. In our previous work, we introduced a novel learning system, called the reservoir of basal dynamics (reBASICS), which features a modular neural architecture (small-sized random neural networks) capable of reducing chaoticity of neural activity and of producing stable self-sustained limit cycle activities. The integration of these limit cycles is achieved by linear summation of their weights, and arbitrary time series are learned by modulating these weights. Despite its excellent learning performance, interpreting a modular structure of isolated small networks as a brain network has posed a significant challenge. Here, we investigate how local connectivity, a well-known characteristic of brain networks, contributes to reducing neural system chaoticity and generates self-sustained limit cycles based on empirical experiments. Moreover, we present the learning performance of the locally connected reBASICS in two tasks: a motor timing task and a learning task of the Lorenz time series. Although its performance was inferior to that of modular reBASICS, locally connected reBASICS could learn a time series of tens of seconds while the time constant of neural units was ten milliseconds. This work indicates that the locality of connectivity in neural networks may contribute to generation of stable self-sustained oscillations to learn arbitrary long-term time series, as well as the economy of wiring cost.

## Introduction

The brain is a complex network of structurally connected and functionally interacting neural units. The network structure is essential for collective computational capability of the neural units. Investigating neural mechanisms from structure to computation is critical to neuroscience and can provide insights into improving artificial neural network systems.

Reservoir computing, a type of artificial recurrent neural network^1–3^, has been utilized to study neural information processing in brain networks^4–6^. Standard reservoir computing uses a fixed, randomly connected reservoir network that creates complex dynamics induced by inputs. The system outputs are obtained through readouts from network units, and only the readout weights are modified so that the outputs produce a target time series. To explore the effects of network structures on reservoir computing, reservoir networks based on brain-like topological features^4,7,8^ or the human connectome^4–6^ have been constructed. These studies have reported that such network structures enhance the learning performance of reservoir computing.

Recently, Kawai et al.^9^ proposed a new type of reservoir computing called the reservoir of basal dynamics (reBASICS), which comprises multiple modules of small random neural networks. If recurrent weight gain is large, random networks with a large size (number of neural units) exhibit chaotic behavior^10^, leading to learning failure because of orbital instability, but a small network size reduces the chaoticity of network activity and generates limit cycles, that is, oscillatory activity, even if the gain is large^11^. They found that the limit cycles from isolated small network modules show a wide frequency spectrum and are orthogonal to each other, creating an orthogonal basis^9^. reBASICS learns to reproduce a target time series as a linear summation of these limit cycles. It exhibits excelled learning performance because of its orthogonal basis. reBASICS indicates the computational role of network modularity, which is a significant feature of brain networks^12–14^. However, the modules in reBASICS are completely isolated, that is, no intermodular connectivity, which has not been observed in the brain networks which have seamless spatial connectivity. Therefore, the contribution of other network structures to reBASICS computation must be explored.

This study investigates the role of locality of connections in brain networks, which are known to be composed of a substantial number of short-range local connections and relatively few long-range connections^15–19^. Such connectivity results in a small-world topology^20–23^, which is supposed to reduce wiring costs^24,25^. Kawai et al.^4^ introduced a small-world topology to a reservoir network to study the impact of local connectivity on computational performance. The study found that the local connectivity suppresses chaoticity and allows for learning, even for large recurrent weight gain that would otherwise cause chaos in a randomly connected standard reservoir. However, the neural activity generated by local connectivity remains unclear. If the neural activities exhibit stable and orthogonal limit cycles to each other, they can be employed for reBASICS computation. The local connectivity structure is expected to bolster the biological plausibility of reBASICS, thus deepening our understanding of the computational roles of local connections in the brain.

Local connectivity has been used to improve the learning performance of reservoir computing. For instance, Rodan and Tino^26,27^ proposed a cyclic reservoir, where neural units are arranged in a cycle and connected one way to the neighboring unit in a cyclic manner. This topology allows input information to be stored in the cycle, providing a large memory capacity. Appeltant et al.^28^ implemented reservoir computing on a single nonlinear unit with delayed self-feedback, which can be considered as a cycle reservoir, using virtual units. Dale et al.^29^ evaluated the computing quality of reservoir networks with a ring, lattice, or torus topologies. They concluded that topological constraints, like the ring, often exhibit limited behavior. These approaches assume that neural units are driven by external inputs and their activities asymptotically depend on the input time series, which is called the echo state property^1^. Further, in these studies, the local connectivity has not been intended to reduce the chaoticity of unit activities.

The essence of reBASICS is the self-organization of mutually orthogonal self-sustained neural oscillations. While conventional reservoir computers assume that neural units are powered by external inputs, reBASICS can autonomously generate time series without continuous input. In our previous study^9^, we demonstrated the effectiveness of a modular structure for reBASICS computation. Now, we suggest that this can similarly be achieved with local connections. To examine the effect of locally connected neural networks on network activity and self-sustained limit cycles in reBASICS computations, we conducted computer simulations on a locally connected reBASICS. Neural units were arranged in a one-dimensional ring or two-dimensional lattice and locally connected with to one another. Our experiment demonstrated that the locally connected neural network generates diverse and stable limit cycles, which are moderately orthogonal to each other. Subsequently, we evaluated the learning performance of locally connected reBASICS in a motor timing task and learning task of the Lorenz time series. The study results indicate that it can learn long time series of tens of seconds, but its performance is inferior to that of the existing modular reBASICS. Nevertheless, the results suggest that the locality of connectivity in neural networks may contribute to neural computation beyond reducing wiring cost.

## Results

### Locally connected reBASICS

**Figure 1:**
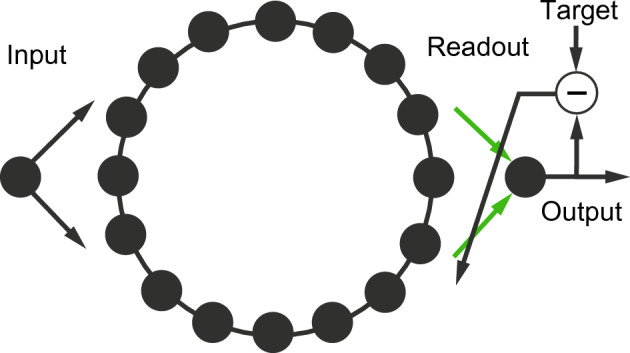
One-dimensional locally connected reservoir of basal dynamics (1D reBASICS). Each neural unit arranged on a lattice has only its neighboring units. The readout output is obtained by a linear sum of units’ activation, in which the weights are trained with the recursive least squares (green arrows).

**Figure 2:**
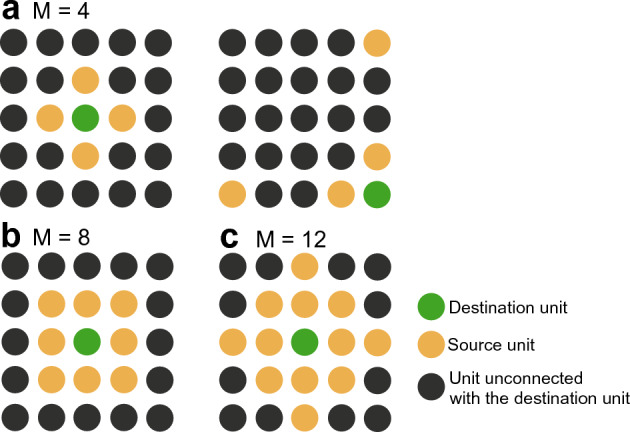
Local connection patterns in the 2D reBASICS. Green destination unit receives connections from yellow neighboring source units. The model with three parameter settings [(**a**) $M = 4$, (**b**) 8, and (**c**) 12] is examined in the experiments. The right panel in (**a**) shows that one end of the lattice was connected to the opposite end.

The locally connected reBASICS comprises *N* neural units arranged in a one-dimensional ring (see Fig. 1) or two-dimensional lattice pattern (see Fig. 2). Hereafter, one-dimensional and two-dimensional locally connected reBASICSs are referred to as 1D and 2D reBASICSs, respectively. Each unit has *E* connections with its neighboring units, with incoming connections from *E* units randomly chosen from *M* units before and after the destination unit in the 1D reBASICS (where *M* is a neighbor parameter ($E \le M$)), and from *M* units in the vicinity of the destination unit in the 2D reBASICS (where $E = M$) (as shown Fig. 2). The connection weights between units are randomly chosen from a Gaussian distribution with zero mean and standard deviation $g / \sqrt{E}$, where *g* is the scaling coefficient for recurrent weights. This local connectivity is expected to reduce chaoticity of network activity and generate limit-cycle time series.

All units receive input signals, and some units emit outputs to the readout unit. The readout weights (represented by green arrows in Fig. 1) are modulated using recursive least squares^30^ to minimize the errors between the readout and target signals. Thus, the weighted summation of various limit-cycle time series can reproduce the target time series. When the limit cycles are orthogonal to each other, they constitute an orthogonal basis, resulting in excellent time-series reproduction capabilities. This computational principle is common to the Fourier and wavelet transformations, which utilize trigonometric functions as their basis.

### Network dynamics

**Figure 3:**
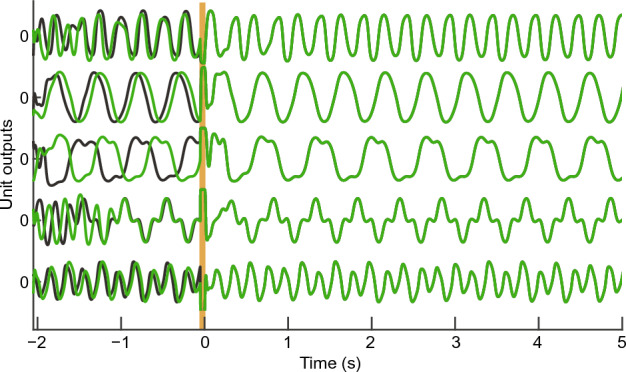
Time series of five sample unit outputs. The green and black curves indicate two runs with different initial unit states. The yellow area between $-51$ to 0 ms indicates the period during which the input pulse was given.

Fig. 3 shows five instances of unit output time series in a 1D reBASICS with $N=50,000$, $E = 10$, and $M = 20$. The trajectories display a range of limit cycles with different frequencies and waveforms. To learn the target time series, a single short input pulse with 50 ms width was introduced into all units at just before 0 ms as a start signal (yellow thick line). This input pulse synchronized the phases of the unit outputs in different runs coping with varying initial conditions. This eliminated the initial state dependency and allowed the network to generate the same limit cycle trajectories across trials.

**Figure 4:**
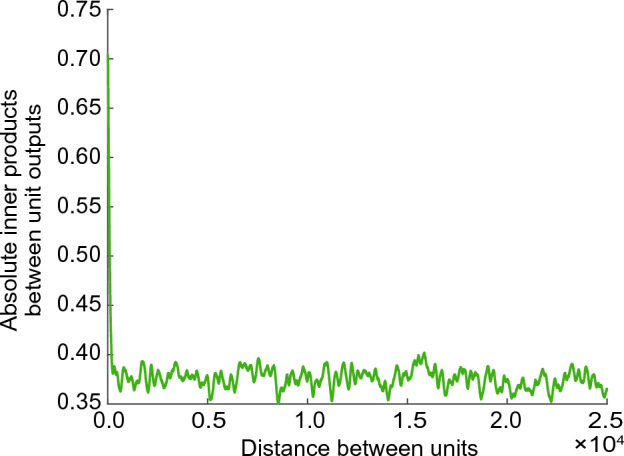
Averaged absolute inner products between unit outputs versus the distance between units. The distance between adjacent units was considered to be 1.

Further, we analyzed the orthogonality of the unit outputs. Fig. 4 shows the average absolute inner product as a function of the distance between units. The inner products between the outputs of adjacent units were large (approximately 0.7), indicating low orthogonality. Interestingly, even for short distances between nonneighbor units, the orthogonality between their outputs increased (approximately 0.37). However, even over longer distances between units, their inner products did not approach to zero.

**Figure 5:**
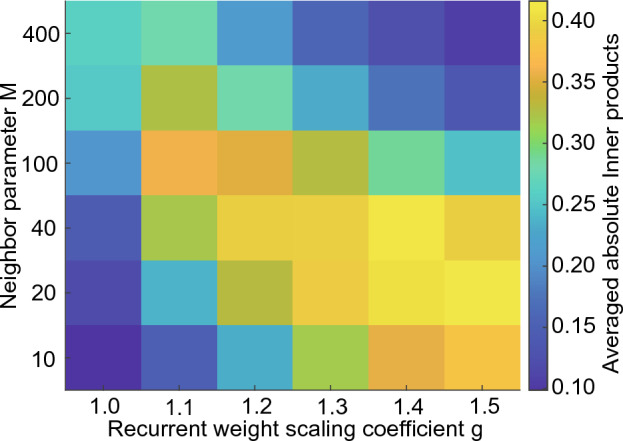
Orthogonality between unit outputs. Colors indicate the averaged absolute inner products between unit outputs, which were averaged over 20 networks. A low value (blue) indicates a high degree of orthogonality.

Fig. 5 illustrates the orthogonality of the network activities of the 1D reBASICS for various values of the neighbor parameter *M* and scaling coefficient *g* for recurrent weights. Evidently, the two highly orthogonal regions are found, where *M* and *g* are low (bottom left) and high (top right), respectively. The former region is more restricted in terms of the locality of the connections and has smaller weights. The latter region has relatively more global connections and larger weights.

**Figure 6:**
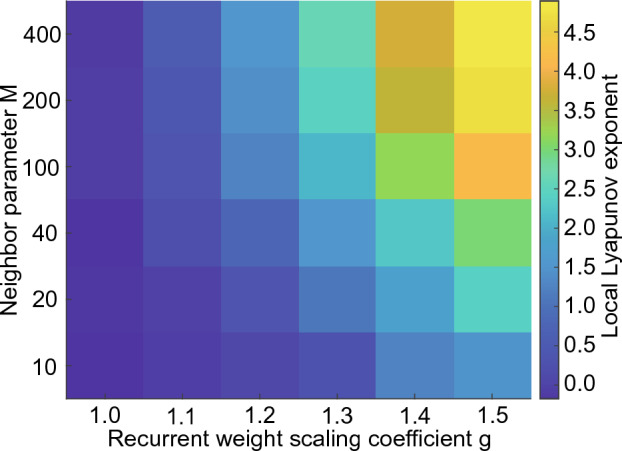
Instability of network activity. Colors indicate the local Lyapunov exponents of unit outputs, which were averaged over 20 networks. A high value (yellow) indicates a high degree of instability.

To evaluate the instability of the network activities, we computed the local Lyapunov exponents (LLE) of the 1D reBASICS in terms of *M* and *g* (Fig. 6). We found that a larger *g* resulted in a higher instability, implying that the network activity became more chaotic. Conversely, a smaller *M* caused a lower instability, indicating that the locality of connectivity could suppress the chaoticity of the network activity. Based on the findings from the orthogonality analysis (Fig. 5), it can be inferred that the area in the top-right quadrant (Fig. 6), which exhibited extensive global connections, was characterized by a high degree of chaos despite being highly orthogonal.

**Figure 7:**
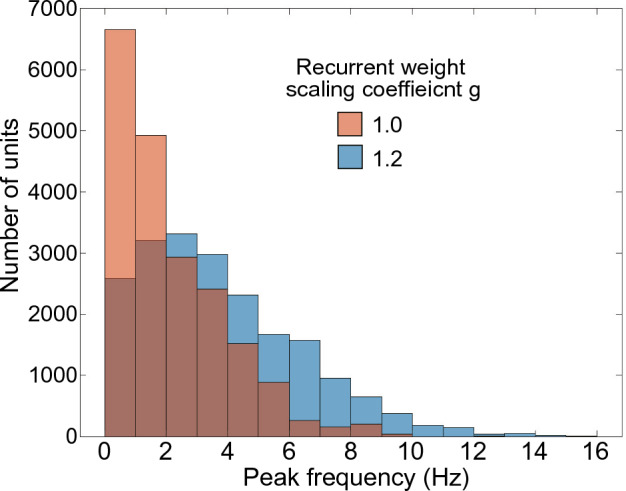
Histograms of peak frequencies of power spectra of unit activities. The networks had 1.0 (orange) and 1.2 (cyan) of recurrent weight scaling coefficient *g* and 20 of neighbor parameter *M*.

**Figure 8:**
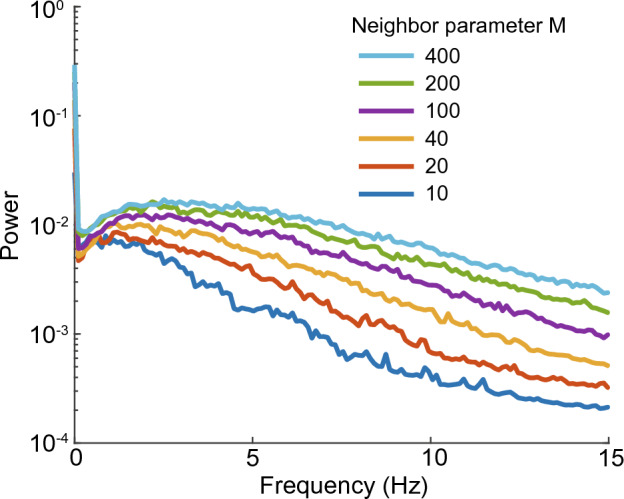
One-sided power spectra of the time series of unit outputs, which were computed using fast Fourier transformation. They are averaged power spectra over all the output units and over 20 networks.

To synthesize an arbitrary time series, time series of various frequencies are required. Thus, we calculated the frequency spectra of the unit outputs of the 1D reBASICS by fast Fourier transformation. Fig. 7 shows histograms of peak frequencies of power spectra when $g = 1.0$ and 1.2 when $M = 20$. We found that a lower *g* resulted in low frequency peaks, reducing frequency variation. Fig. 8 shows the frequency spectra of unit outputs for various *M*s when $g = 1.2$. The output units with a large *M* showed a wide range of power spectra, and a small *M* shows low power in high frequencies. The orthogonality analysis (Fig. 5) indicates that the area in the bottom-left quadrant, where *g* and *M* are small, was characterized by low frequency band despite being highly orthogonal.

### Motor timing task

The motor timing task is commonly used to assess the learning ability of reservoir computing with self-sustained neural activity^9,31,32^. In this task, the input to the system is a single pulse from $-51$ ms to 0 ms, while the desired output is a single Gaussian pulse with a peak after a specific interval from the end of the input pulse. Owing to the interval period without input and output, previous studies have demonstrated that standard reservoir computing including echo state networks and FORCE^33^ cannot complete this task successfully^9,31^.

**Figure 9:**
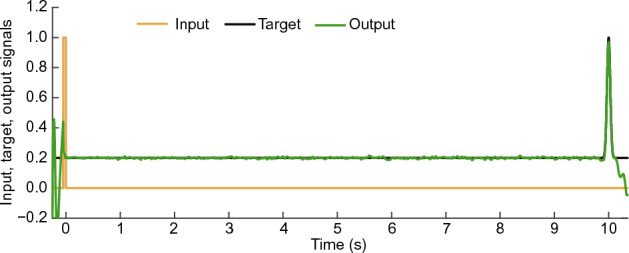
An example of the readout output of locally connected reBASICS after learning for interval of 10 s.

An example of the readout output of a 1D reBASICS in a test trial with a 10 s interval is shown in Fig. 9. The readout output successfully replicated the Gaussian pulse at 10 s after the input pulse. Notably, despite the time constant of neural units ($\tau = 10$ ms), it was capable of learning at intervals of one minute ($R^2=0.50$) and two minutes ($R^2=0.25$).

**Figure 10:**
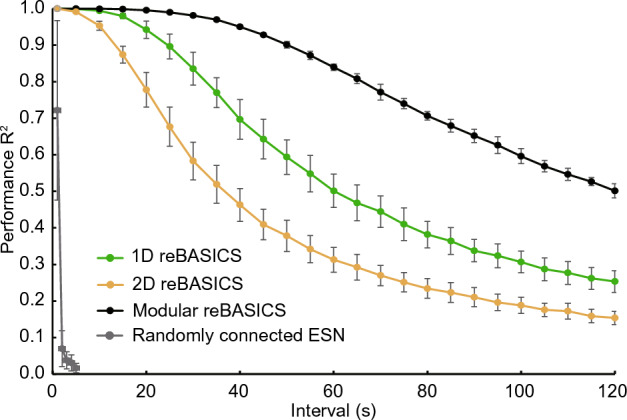
Performance up to 120 s of intervals. The performance was averaged over 20 networks (mean ± s.d.). The green, yellow, black, and gray curves indicate the performance of 1D and 2D reBASICSs, respectively, modular reBASICS^9^, and randomly connected echo state networks (ESNs) respectively.

We evaluated the learning performance using the coefficient of determination (the squared Pearson correlation coefficient) $R^2$ between the system output and target signal. As shown in Fig. 10, we varied the intervals parametrically and plotted $R^2$. The performances of the 1D reBASICS and modular reBASICS^9^ are indicated by the green and black curves, respectively. Although the performance of the 1D reBASICS was inferior to that of the modular reBASICS, it was capable of learning even at very long intervals of one minute or more.

Conventional reservoir computers (represented by randomly connected echo state networks: ESNs, depicted by the gray curve), typically driven by external input, faltered in the timing task due to an interval without input. These ESNs had a network size of 1,000 and a spectral radius of 1.0. Similar results were observed when the network size increased. This finding aligns with existing studies^9,31^.

**Figure 11:**
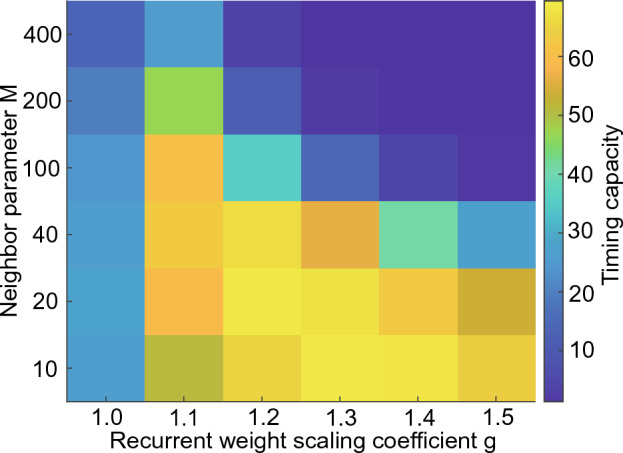
Timing capacity of 1D reBASICS. It was averaged over 20 networks.

Fig. 11 shows the timing capacity, defined as the area under the $R^2$ curve up to 120 s, of the 1D reBASICS in terms of *M* and *g*. The best timing capacity was obtained when $M = 20$ and $g = 1.2$. Conversely, the timing capacity decreased when *M* and *g* (top right region) were large, and when $g = 1.0$ (left column).

**Figure 12:**
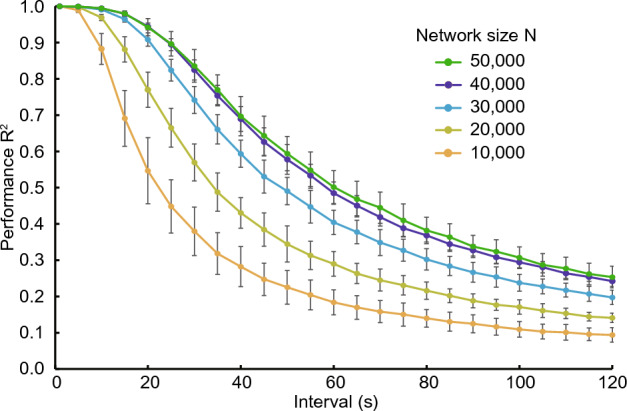
Performance of 1D reBASICS with various network sizes. It was averaged over 20 networks (mean ± s.d.).

Fig. 12 shows the performance of 1D reBASICS with various network sizes *N*. The green curve at $N = 50,000$ is the same as that in Fig. 10. This curve does not differ from the curve for $N = 40,000$, indicating that the performance was saturated at $N = 50,000$. Reducing *N* resulted in a significant decrease in the performance. However, a small network size does not mean that learning fails; for example, at $N = 10,000$, the performance for a 10-second interval was $R^2=0.88$.

**Figure 13:**
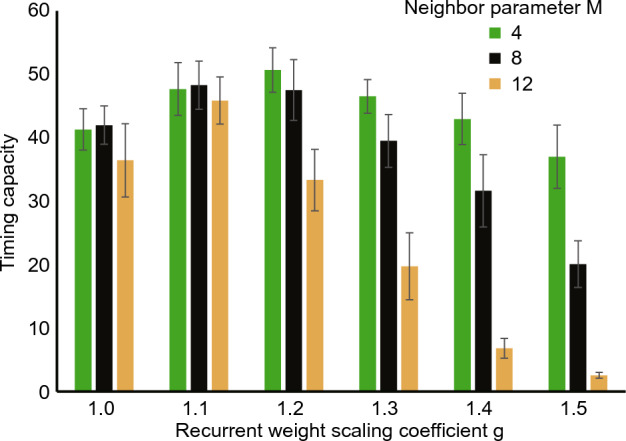
Timing capacity of 2D reBASICS. It was averaged over 20 networks.

In Fig. 10, the yellow curve depicts the result of a 2D reBASICS with $M = 4$. The figure shows that the performance of the 2D reBASICS was lower than that of the 1D reBASICS. Fig. 13 shows the timing capacity of 2D reBASICS with $M = 4$ (green), 8 (black), and 12 (yellow). The best timing capacity was obtained when $M = 4$ and $g = 1.2$.

### Learning of the Lorenz time series

**Figure 14:**
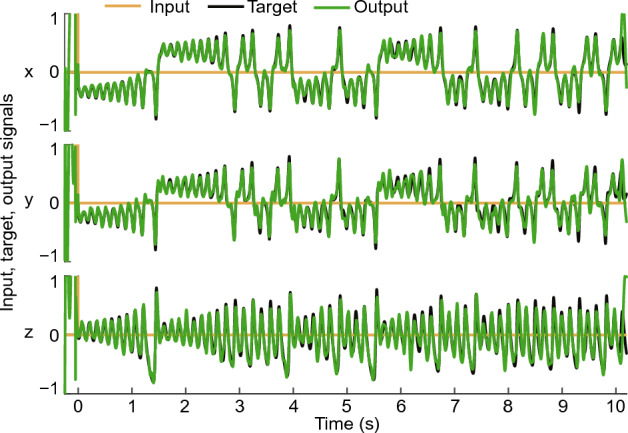
Learning the chaotic trajectories in the Lorenz system. The time series show an example of the system output (green), target signal (black), and input signal (yellow) after 1D reBASICS learned the Lorenz system for 10 s.

Fig. 14 presents an instance of the learning outcomes of the 1D reBASICS when applied to the Lorenz time series for a period of 10 s. Similar to the previous experiment, the input consisted of a single pulse at time 0 (yellow lines in Fig. 14). Following the input, the self-sustained limit cycles were able to produce a time series resembling the Lorenz time series. The performance for *x*, *y*, and *z* in the Lorenz system were $R^2 = 0.96 \pm 0.025$, $0.91 \pm 0.031$, and $0.87 \pm 0.031$ (mean ± s.d. for 20 networks), respectively. This result indicates that the 1D reBASICS is capable of learning non-periodic complex time series, such as the Lorenz time series.

## Discussion

The experimental results indicate that local connectivity contributes to the generation of diverse limit cycles with less chaotic behavior. This facilitates reBASICS computation, which involves reproducing time-series through weighted sums using these limit cycles as a basis. The self-sustained nature of these limit cycles allows them to perform a motor timing task with a long interval without requiring any inputs.

To maximize the learning performance of the locally connected reBASICS, a balance between the stability and diversity of unit activities was crucial. Large values of *M* and *g* resulted in chaotic behavior, leading to a breakdown in learning. Conversely, smaller values of *M* and *g* caused less variation in the frequency of the limit cycles of unit activities. Therefore, it was essential to generate a range of self-sustained activities to a degree that would prevent chaos. This asymptotically stable regime is referred to as the “edge of chaos” in reservoir computing^34,35^. In this study, we utilized the concept of “oscillatory edge of chaos” and found that the degree of the locality of connections was a crucial parameter in determining the edge, as well as the weight gain. Additionally, the model necessitated significantly more neural units (network size *N*) than conventional reservoir computers. When *N* was reduced from 40, 000 to a lower value, the performance markedly declined. This suggest that a sizable network is essential to produce diverse (orthogonal) neural oscillations.

The learning performance of the locally connected reBASICSs was found to be inferior to that of the modular reBASICS, which could be attributed to the insufficient orthogonality between unit activities. Although the units were distant from each other owing to local connectivity, their activities were weakly correlated because they were not completely separate. Local connectivity and modular structures are typical features of brain networks. By combining these features, the reBASICS was expected to exhibit stable and high performance over a wide range of parameters.

The reBASICS was originally proposed as a general model of either the corticostriatal system or the cerebellum, two hypotheses. The fact that local connectivity enables reBASICS computations in both hypotheses supports this view. In the former model, the reservoir network and readout learning were interpreted as the cortical network and dopamine modulation in the striatum, respectively. In another study, we demonstrated that timing learning could be achieved even if the supervised learning of reBASICS readouts was replaced by reward-modulated Hebbian learning^36^. As it is well known, the majority of cortical networks consist of local connections in small-world brain networks^17–19^. However, considering the existence of long-range connections in cortical networks, it is necessary to investigate whether reBASICS with such connections will work well. In the latter cerebellar model, the reservoir network and readout learning were interpreted as the granular layer (a network of granule cells and interneurons) and Purkinje cell plasticity, respectively. This model is based on the cerebellar reservoir hypothesis^37–39^. Tokuda et al.^39^ proposed a biologically detained cerebellar model in which Golgi cells, which are interneurons in the granular layer, were mutually connected via gap junctions. Owing to the connectivity being local, this anatomical fact is consistent with the structure of the locally connected reBASICS.

The locality of connectivity in neural networks is supposed to be due to the wiring economy, that is, shorter axons are less costly^24,25^. In addition to the economic brain view, we emphasize the computational role of the local connectivity in neural networks, which includes suppressing the chaoticity of network activity and generating diverse stable oscillations to learn arbitrary time series. These findings lead to a better understanding of neural information processing and the invention of better recurrent neural networks.

## Conclusion

In this study, experiments conducted using a locally connected neural network revealed that local connectivity reduced the chaoticity of network activity and produced stable limit cycle activities. Leveraging oscillatory activities, we proposed a locally connected reBASICS model that integrated the activities to approximate the target time series. The self-sustained nature of the activities enabled the system to learn the timing within an interval period without input. Additionally, we demonstrated that the locally connected reBASICS was capable of accurately learning complex time series, including the Lorenz system. Importantly, we found that local connectivity and modular structure are critical to reBASICS neural computation, both of which are prominent features of brain networks. Our work bridges the gap between function/structure and computation of neural networks and provides a better understanding of temporal processing and motor learning in the brain.

## Method

The dynamics of state $x_i(t)$ of unit *i* ($i = 1, 2, \dots , N$) at time *t* are represented as a firing rate model:

1 $$\begin{aligned} \tau \frac{\textrm{d}x_i(t)}{\textrm{d}t} = -x_i(t) + \sum _{j=1}^N W_{ij}^{\textrm{Rec}} r_j (t) + W_i^{\textrm{In}} u(t) + I_i^{\textrm{Noise}}, \end{aligned}$$

where $r_i(t) = \tanh (x_i(t))$ represents the activity level of a unit *i*. *N*, $\tau$, and *u*(*t*) denote the network size, time constant, and input signal, respectively. $W_i^{\textrm{In}}$ and $I_i^{\textrm{Noise}}$ denote the input weight and noise term, respectively, which are drawn from Gaussian distributions with zero mean and s.d of one and s.d. $I_0$. $W_{ij}$ denotes the connection weight from units *j* to *i*. If there exists a connection from *j* to *i*, the value of $W_{ij}$ is randomly chosen from a Gaussian distribution with zero mean and s.d., $g / \sqrt{E}$, where *g* and *E* denote the recurrent weight scaling coefficient and number of incoming connections, respectively; otherwise, $W_{ij} = 0$.

If the topology of the recurrent network is random, a high-gain regime with a large *g* ($> 1.0$) makes the network activity self-sustained, but chaotic^10^. Although modular reBASICS sets $g > 1.0$ to generate self-sustained activity, using a small network size enables small network modules to output stable orbits, such as limit cycles^9^. Similarly, in locally connected reBASICS, using local connectivity is expected to reduce the chaoticity of network activity, even when $g > 1.0$, and generate limit cycles with orbital stability. Some unit activities do not exhibit limit cycles and converge at a fixed point. Because such an activity is unnecessary for learning, we randomly select *L* units from those that do not converge to a fixed point and remain active, and regard them as output units *k* ($k \in O$, where *O* is a set of indices of output units and $|O| = L$). Specifically, units with a difference of 0.01 or more between the maximum and minimum activity from 5 s to the learning end time were considered active units. The value of this threshold was empirically determined. In the standard instance of 1D reBASICS depicted in Figs. 9 and 10, 21,895 units (44%) were disregarded as they had converged. If the number of active units dropped below *L*, the simulation was restarted from the outset, a scenario not observed in the experiments.

These oscillatory activities of the output units $r_k(t)$ are converted to the readout output *y*(*t*) by a linear sum given as

2 $$\begin{aligned} y(t) = \sum _{k \in O} W_k^{\textrm{Out}} (t) r_k(t), \end{aligned}$$

where $W_k^{\textrm{Out}}$ is the readout weight trained using the recursive least squares method. Let $\textbf{W}^{\textrm{Out}} (t)$ and $\textbf{r} (t)$ denote the weights and activities of the output units, respectively, as vectors of length *L*. $\textbf{W}^{\textrm{Out}} (t)$ is updated at time *t* as follows:

3 $$\begin{aligned} \textbf{W}^{\textrm{Out}} (t)= & {} \textbf{W}^{\textrm{Out}} (t - \Delta t) - e(t) \textbf{P} (t) \textbf{r}^{\top } (t), \end{aligned}$$

4 $$\begin{aligned} e(t)= & {} \textbf{W}^{\textrm{Out}} (t - \Delta t) \textbf{r}^{\top } (t) - d (t), \end{aligned}$$

where *d*(*t*) denotes the target time series and $\textbf{P}(t)$ is an $L \times L$ matrix that corresponds to the running estimate of the inverse sample covariance matrix of $\textbf{r} (t)$^30^. $\textbf{P}(t)$ is updated as follows:

5 $$\begin{aligned} \textbf{P}(t) = \textbf{P}(t - \Delta t) - \frac{\textbf{P} (t - \Delta t) \textbf{r}^{\top }(t) \textbf{r}(t) \textbf{P} (t - \Delta t)}{1 + \textbf{r} (t) \textbf{P} (t - \Delta t) \textbf{r}^{\top } (t)}. \end{aligned}$$

The initial value of $\textbf{P} (t)$ is set to $\textbf{P}(0) = (1/\alpha ) \textbf{I}$, where $\textbf{I}$ denotes an identity matrix and $\alpha$ is a constant.

### Experimental settings

The numerical solutions of Eq. (1) were obtained using the Euler method with a simulation step size of 1 ms. The recursive least squares method was applied once every two steps in the training period, and $\Delta t$ in Eqs. (3)–(5) is set to 2 ms. The simulation begins at time $-250$ ms ($-2050$ ms in the simulation in Fig. 3). The initial state of each unit was set to a uniform random value in the range $[-1, 1]$. In all the experiments, the input *u*(*t*) was a single pulse with a magnitude of five between $-51$ and 0 ms and zero in other periods. This single-pulse input was used to initialize the phases of the limit cycles of the unit activities, that is, to reduce the dependency on the initial unit states (see Fig. 3).

Unless otherwise stated, the parameter values listed in Table 1 were used in the 1D reBASICS. The parameters *M* and *g* were empirically determined as optimal values based on the experimental results presented in Fig. 11. The value of *N* was determined by the experimental results shown in Fig. 12, where the performance was approximately saturated at that value. For a valid comparison with modular reBASICS^9^, the parameters *L*, *E*, $\tau$, $I_0$, and $\alpha$ were matched with the proposed method. Therefore, the modular reBASICS had 500 modules consisting of 100 units, and each module had two output units, resulting in total 1,000 output units.

**Table 1:** Parameter values.

Parameter	Description	Value
*N*	Network size (the number of units)	50,000
*M*	Neighbor parameter	20
*L*	The number of output units	1,000
*g*	Recurrent weight scaling coefficient	1.2
*E*	The number of in-coming connections	10
$\tau$	Time constant	10 (ms)
$I_0$	Noise amplitude	0.001
$\alpha$	Initial value for recursive least squares	1.0

In the 2D reBASICS, *N* was set to 52,900 ($230 \times 230$ lattices), where one end of the lattice was connected to the opposite end. We considered three parameter values for the neighbor parameter: $M = 4$, 8, or 12 (see Fig. 2), and the number of connections was set to $E = M$. The other parameter values were the same as those used in the 1D reBASICS.

#### Evaluation

For all experiments, the coefficient of determination (the squared Pearson correlation coefficient) $R^2$ between the system output *y*(*t*) and target *d*(*t*) from 1 ms to the end of a task was used to evaluate the learning performance. This value was averaged over 10 test trials and over 20 different networks.

To evaluate the instability of network activity, we used LLE, which was estimated in a similar manner as in references^9,31,32^. A small perturbation is given to all units at time 0 ms: $\textbf{x}^{\textrm{pert}}(0) = \textbf{x}(0) + \epsilon$, where $\textbf{x} (t) = \{ x_{1}, x_{2}, \dots , x_{N} \}$ and $\epsilon = 10^{-5}$. The logarithm of the distance between the unperturbed and perturbed trajectories was then computed over time, which was normalized by the initial distance at 0 ms.

6 $$\begin{aligned} \textrm{dist} (t) = \log \left( \frac{\Vert \textbf{x}^{\textrm{pert}} (t) - \textbf{x} (t) \Vert }{\Vert \textbf{x}^{\textrm{pert}} (0) - \textbf{x} (0) \Vert } \right) . \end{aligned}$$

The log distance $\textrm{dist} (t)$ was averaged over 10 trials with different initial unit states. The LLE was defined as the slope of the function from 1 s to 10 s. If LLE $>0$, the system is unstable, and the larger the LLE, the more unstable (chaotic) the system is.

We evaluated the orthogonality of network activity as the average absolute inner products between unit outputs. The temporal vectors of the time series $r_{i} (t)$ from $t = 1,000$ to 10, 000 ms were normalized to the unit vectors. The absolute inner products of all combinations of vectors for all units were computed. The inner products were averaged over all combinations and over 20 different networks. A low value of the average absolute inner product indicates a high orthogonality of the entire network activity.

#### Motor timing task

The input was a single pulse at time 0 ms. The desired output was a single Gaussian pulse with a peak at a specific interval from the end of the input pulse. The magnitude and standard deviation of the Gaussian pulse were 1 and 30 ms, respectively. The interval was set from 1 to 120 s in the experiments. The system was trained from time 0 ms to the interval plus 150 ms. The reBASICSs were trained on 10 trials (a trial is a run to the end of the training period), and then their learning performance $R^2$ was evaluated using 10 untrained test trials. We defined the timing capacity as the area under the $R^2$ curve over intervals of up to 120 s.

#### Learning of Lorenz time series

We used the Lorenz system^40^ as the target signal to evaluate the learning performance for complex and unpredictable time series. The Lorenz time series was obtained through interactions between three variables:

7 $$\begin{aligned} \frac{\textrm{d}x(t)}{\textrm{d}t}= & {} -p x(t) + p y(t), \end{aligned}$$

8 $$\begin{aligned} \frac{\textrm{d}y(t)}{\textrm{d}t}= & {} - x(t) z(t) + r x(t) - y(t), \end{aligned}$$

9 $$\begin{aligned} \frac{\textrm{d}z(t)}{\textrm{d}t}= & {} x(t) y(t) - b z(t), \end{aligned}$$

where $p = 10$, $r = 28$, and $b = 8/3$, and we set $x(0) = 0.1$, $y(0) = 0$, and $z(0) = 0$. We obtained the numerical solution for Eqs. (7)–(9), using the fourth-order Runge–Kutta method with a step size of 0.001. Then, the time series was downsampled to 1/5 of its length and normalized to $[-1, 1]$ in magnitude, resulting in a 10 s three-dimensional target signal, where 1 ms was regarded as one step. As the motor timing task, the input was a single pulse of 50 ms. The 1D reBASICS had three readouts corresponding to the *x*, *y*, and *z* variables of the Lorenz time series. Each readout weight was modulated using the corresponding target time series. The number of trials for training and testing was the same as that for the learning of motor timing.

## Data Availability

The datasets used and/or analysed during the current study available from the corresponding author on reasonable request.
